# Sequence analysis of nonulosonic acid biosynthetic gene clusters in *Vibrionaceae* and *Moritella viscosa*

**DOI:** 10.1038/s41598-020-68492-3

**Published:** 2020-07-20

**Authors:** Marie-Josée Haglund Halsør, Bjørn Altermark, Inger Lin Uttakleiv Ræder

**Affiliations:** The Norwegian Structural Biology Centre (NorStruct), Department of Chemistry, UiT- The Arctic University of Norway, 9037 Tromsø, Norway

**Keywords:** Carbohydrates, DNA, Enzymes, Metabolomics, Molecular modelling

## Abstract

Nonulosonic acid (NulO) biosynthesis in bacteria is directed by *nab* gene clusters that can lead to neuraminic, legionaminic or pseudaminic acids. Analysis of the gene content from a set mainly composed of *Aliivibrio salmonicida* and *Moritella viscosa* strains reveals the existence of several unique *nab* clusters, for which the NulO products were predicted. This prediction method can be used to guide tandem mass spectrometry studies in order to verify the products of previously undescribed *nab* clusters and identify new members of the NulOs family.

## Introduction

**Figure 1:**
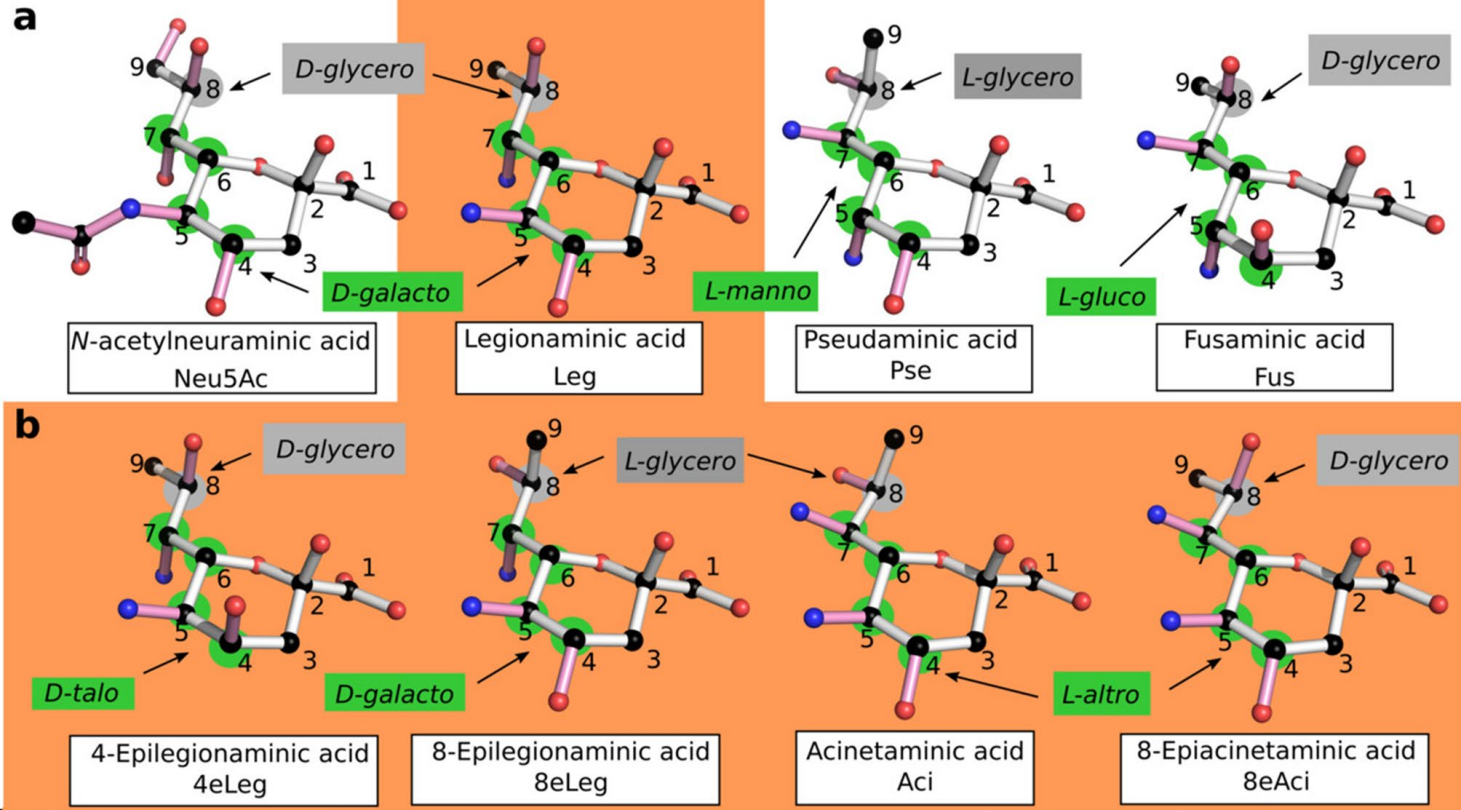
Structure of known NulOs. The common, nine-carbon backbone of NulOs is represented with white bonds, and the carbons are numbered. The absolute configuration of each isomer is indicated in either gray or green depending on the concerned chiral centers, which are marked by disks of the corresponding color. (**a**) The three main NulOs families, according to their synthesis pathways, and the newly identified fusaminic acid (Fus). Neuraminic acid (Neu) is represented carrying an *N*-acetyl group in C5 (Neu5Ac), since it is the most commonly found species. Legionaminic (Leg), pseudaminic (Pse) and Fus carry N-linked groups in C5 and C7, which were omitted for clarity leaving only the nitrogen atom. (**b**) Isomers from the legionaminic acid synthesis pathway presenting different absolute configurations. As for Leg, N-linked groups carried at the C5 and C7 positions are represented by the nitrogen atom only. The orange background serves to highlight the common synthesis pathway of Leg isomers. Figure taken from^82^.

Nonulosonic acids (NulOs) are a diverse family of 9-carbon $\alpha$-keto acids present on the cell surface. Of those, neuraminic acids (Neu), a family of dideoxynonulosonic acids better known as sialic acids, are most widely spread in vertebrates in which they were first discovered^1, 2^. The most common bacterial NulOs are tetradeoxynonulosonic acids, although neuraminic acids are also found and one instance of a trideoxy-compound has been reported recently^3, 4^. They are specific to bacteria and are displayed on the bacterial cell surface as parts of the capsule, flagella, lipopolysaccharide and pili^3, 5–9^. The various tetradeoxy-NulOs are somewhat classified according to their absolute configuration, as presented in Fig. 1. Legionaminic acid (Leg, see panel a) is a 5,7-diamino-3,5,7,9-tetradeoxy-d-*glycero*-d-*galacto*-non-2-ulosonic acid, while pseudaminic acid (Pse) is the l-*glycero*-l-*manno* isomer^5, 6, 10, 11^. The l-*glycero*-d-*galacto* and l-*glycero*-d-*talo* isomers are designated as 8- and 4-epilegionaminic acids, respectively^12, 13^. The l-*glycero*-l-*altro* isomer is known as Acinetaminic acid (Aci), and the d-*glycero*-l-*altro* isomer as its 8-epimer^14, 15^. Most recently, the (d/l)-*glycero*-l-*gluco* isomer was identified as fusaminic acid (Fus), like its trideoxy-counterpart^4, 16^.

NulOs from pathogenic bacteria play a central role in host-pathogen interactions. Their structure mimics that of host neuraminic acids, which enables them to bind to the Siglecs (Sialic acid-binding immunoglobulin-type lectins) of host immune cells^17, 18^. This type of interaction results in the inhibition of immune cell activation. As a critical component of flagella and pili they indirectly affect host colonization, but also bacterial motility in general^19–21^. In addition to synthetizing NulOs themselves, bacteria can utilize host NulOs to evade the immune system^22, 23^.

NulOs are synthetized by three main pathways (Nonulosonic Acid Biosynthesis, or NAB)^24^, leading to Neu, Leg and Pse related compounds. The biosynthesis is directed by *nab* gene clusters which harbor homologous genes that are responsible for the main biosynthesis steps^25–27^. Aci is synthetized by a set of additional genes affixed to the Leg cluster, and the gene cluster responsible for the synthesis of Fus has not been investigated yet^14^. The composition and organization of *nab* clusters varies not only between pathways and species, but also within species^24, 28^. Only one gene, coding for the *N*-acetylneuraminate synthase NeuB, is conserved across both pathways and species. The NeuB homologs catalyze the key reaction of NulO biosynthesis, the condensation of a hexosamine precursor with pyruvate leading to either Neu, Leg, or Pse.

*Aliivibrio salmonicida* is a psychrophilic fish pathogen causing cold water vibriosis, a disease that used to seriously impact the output of salmon aquaculture before the use of vaccines^29–31^. It is known to produce mono- and di-*N*-acetylated Neu (Neu5Ac and Neu5Ac7(9)Ac), and the presence of an acetamidino variant of Leg (8eLeg5Am7Ac) has been detected in its lipopolysaccharide^32–34^. *Moritella viscosa* is the main agent in causing the winter ulcer disease in salmon and cod, with *Aliivibrio wodanis* as a co-pathogen^35–37^. As for *A. salmonicida*, Neu5Ac and Neu5Ac7(9)Ac were shown to be produced by *M. viscosa*^38^. The NulO content of *A. wodanis* has not been investigated so far, but its *nab* gene cluster appears similar to that of *Vibrio vulnificus* CMCP6, which produces the alanyl carrying Leg variant Leg5Ac7AcAla^39^. The diversity of *nab* clusters within *Vibrionaceae* has already been shown, with an emphasis on *Vibrio vulnificus*^28^. The distribution of NulOs in *Moritellaceae* has not been studied.

**Table 1:** Overview of bacterial strains.

Organism	Strain	Source	Accession	Reference(s)
*Aliivibrio logei*	MR17-77	*Porifera indet*	This study	^41, 42^
*A. salmonicida*	LFI1238	*Gadhus morhua*	NC_011312.1	^71^
*A. salmonicida*	R8-68	*Eurythenes gryllus*	This study	^41, 42^
*A. salmonicida*	R8-70	*Eurythenes gryllus*	This study	^41, 42^
*A.* sp.	R8-63	*Eurythenes gryllus*	This study	^41, 42^
*A. wodanis*	06/09/139	*Salmo salar*	LN554846.1	^72, 73^
*Moritella viscosa*	06/09/139	*Salmo salar*	LN554852.1	^72, 73^
*M. viscosa*	F57	*Cyclopterus lumpus*	FPLF^a^	^36, 40^
*M. viscosa*	K56	*Salmo salar*	FRDV^a^	^36, 40^
*M. viscosa*	K58	*Salmo salar*	FRDT^a^	^40, 74^
*M. viscosa*	LFI5006	*Salmo salar*	FPLG^a^	^40, 75^
*M. viscosa*	NVI4917	*Oncorhynchus mykiss*	FRDS^a^	^40, 76^
*M. viscosa*	NVI5482	*Gadhus morhua*	FPLE^a^	^40, 76^
*M. viscosa*	Vvi7	*Salmo salar*	FRDU^a^	^40, 76^
*M. viscosa*	Vvi11	*Salmo salar*	FRDQ^a^	^40, 76^
*Photobacterium phosphoreum*	SP005	*Onogadus argentatus*	This study	^41^
*Vibrio anguillarum*	NB10	*Gadhus morhua*	LK021130.1	^77–79^
*V.* sp.	B9-25K2	*Halichondria* sp.	This study	^41^
*V. vulnificus*	CMCP6	*Homo sapiens*	AE016795.3	^80, 81^

^a^Whole genome sequencing (WGS) project identifier.

This study investigates the presence and composition of *nab* gene clusters in a set of strains from *A. salmonicida* (*Vibrionaceae*) and *M. viscosa* (*Moritellaceae*), as well as *A. wodanis* and a few other members of the *Vibrionaceae* family. The strains were previously isolated from different kinds of organisms such as fish, sponge and amphipod (for a summary of the strains used in this study, see Table 1 in the Materials and Methods section). They were screened for the presence of NeuB sequences, which were then used to locate *nab* clusters in their genomes as well as investigate the sequence determinants of NeuB substrate specificity. Each cluster was analyzed in terms of gene content and organization, using sequence homology as a basis for functional annotation. From this, hypotheses pertaining to the nature of the NulO(s) produced by each strain could be made, and a set of candidates for experimental analysis was obtained. The NulO content of a few strains was analyzed using mass spectrometry in order to obtain preliminary experimental results.

## Results and discussion

### Comparison of NeuB sequences

#### Sequence alignment and phylogeny

**Figure 2:**
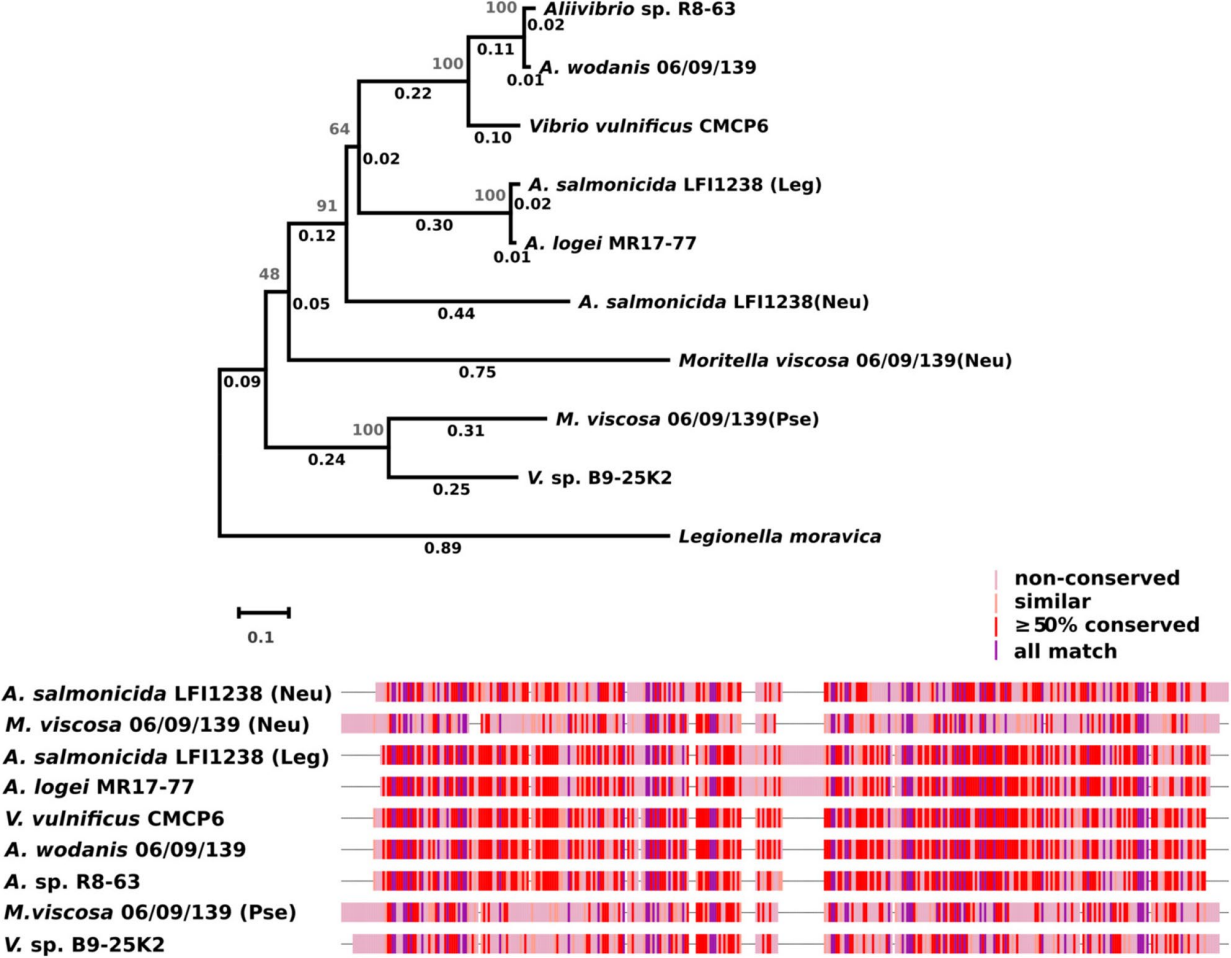
Comparison of unique NeuB sequences. Top: Evolutionary history of NeuB sequences amongst target genomes. The evolutionary history was inferred using the Neighbor-Joining method^59^, with a bootstrap test (500 replicates)^83^. The tree is drawn to scale, with branch lengths (same units as the evolutionary distances used to infer the tree) and bootstrap values shown next to the branches. The evolutionary distances were computed using the Poisson correction method^60^ and are in the units of the number of amino acid substitutions per site. The rate variation among sites was modeled with a gamma distribution (shape parameter = 5). This analysis involved 10 amino acid sequences. All positions containing gaps and missing data were eliminated (complete deletion option). There were a total of 318 positions in the final dataset. Evolutionary analyses were conducted in MEGA X^61^. Bottom: Fingerprint of NeuB sequence alignment. The sequences were aligned using the MUSCLE algorithm^57, 58^. Gaps are shown as “–”.

NeuB protein sequences from *A. salmonicida* LFI1238, *M. viscosa* 06/09/139 and *A. wodanis* were used as queries for conducting a sequence similarity search within the set of target genomes (see Table 1). Out of the 15 targets, 10 had at least one sequence identical to one of the queries, and 3 had a similar sequence. As expected, no NeuB coding sequences were found in either *P. phosphoreum* or *V. anguillarum*. A fingerprint of the alignment of unique NeuB sequences and NeuB phylogeny are presented in Fig. 2 (the full alignment is included as Supplementary Information, in Figure S1).

**Figure 3:**
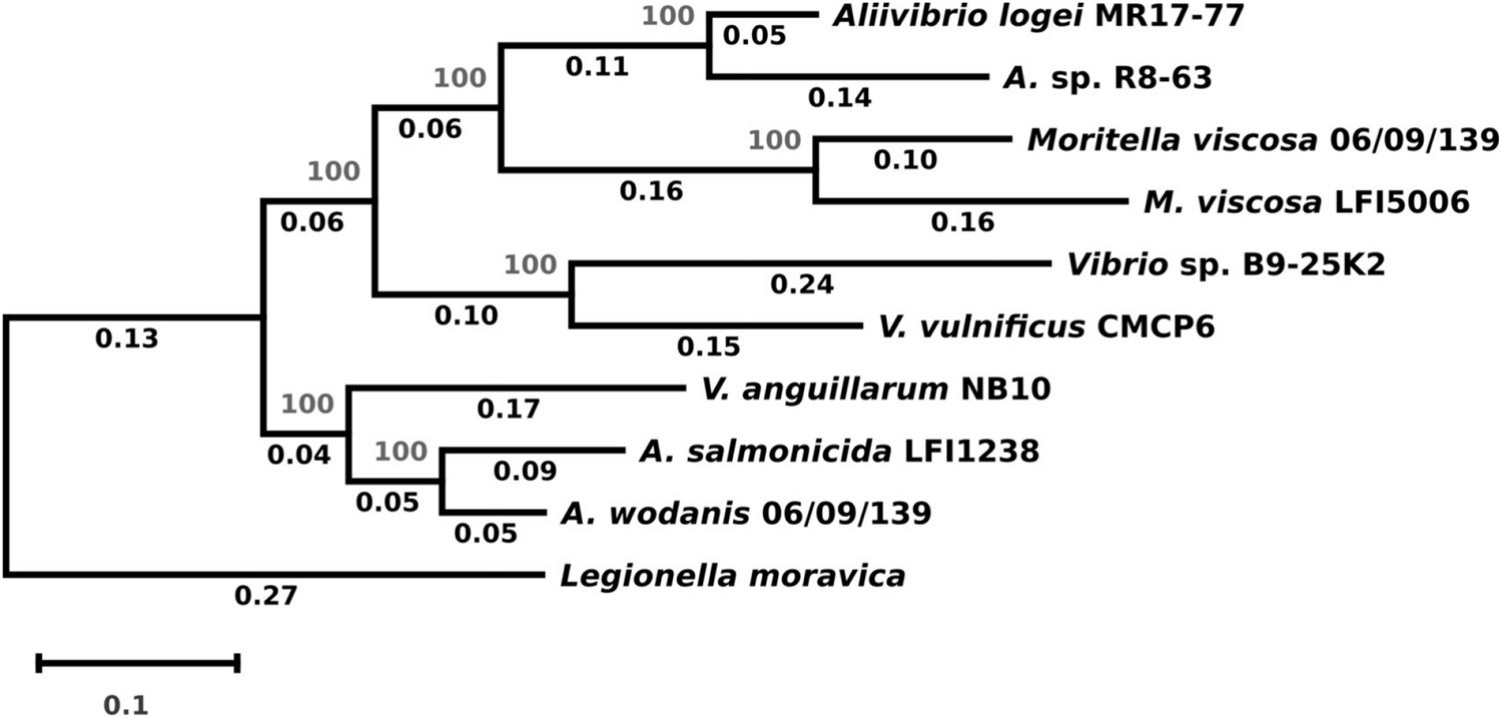
Evolutionary history for *Vibrio* sp. B9-25K2. The tree was built in MEGA X using the Neighbor-Joining method^59^. The unrooted, optimal tree with the sum of branch length = 2.12420929 is shown. The percentage of replicate trees in which the associated taxa clustered together in the bootstrap test (1000 replicates) are shown next to the branches^83^. The evolutionary distances were computed using the Jukes–Cantor method^62^ and are in the units of the number of base substitutions per site. The rate variation among sites was modeled with a gamma distribution (shape parameter = 5). This analysis involved 10 nucleotide sequences. All positions containing gaps and missing data were eliminated (complete deletion option). There were a total of 8050 positions in the final dataset. Evolutionary analyses were conducted in MEGA X^61^.

**Figure 4:**
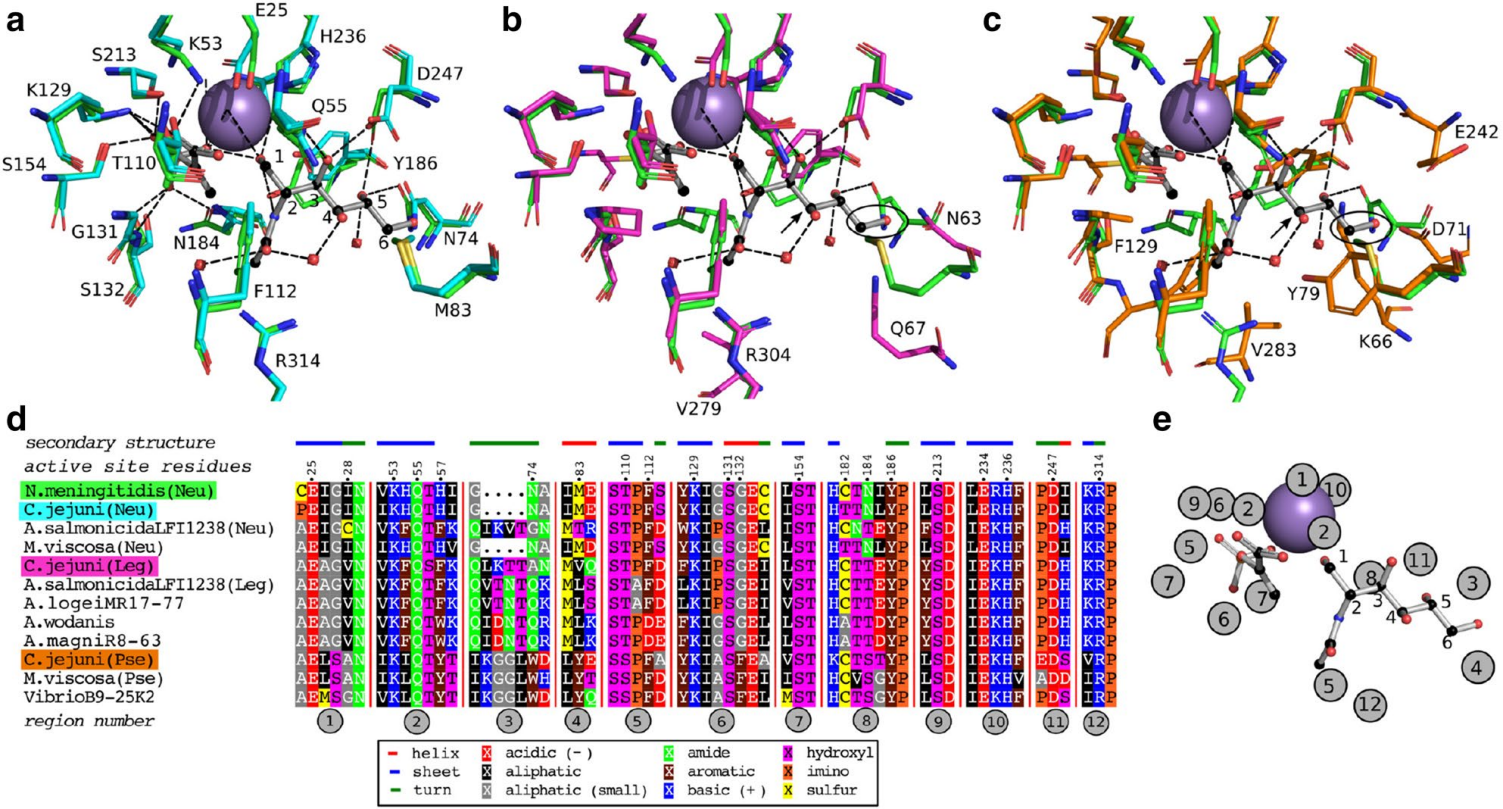
Comparison of NeuB active sites of *C. jejuni* and *N. meningitidis* with the unique NeuB sequences from *Aliivibrio*, *Vibrio*, and *Moritella viscosa*. (**a**)–(**c**) Sequence structure comparison of NeuB homologs from *Neisseria meningitidis* (nmNeuB, green backbone, PDB ID: 1xuz) and *Campylobacter jejuni* (cjNeuB1-3, in cyan, pink and orange). Ball and stick representations: reduced ManNAc (rMAnNAc, right) and phosphoenol pyruvate (PEP, left). Purple sphere: Mn$^{2+}$. (**a**) Neu pathway. The carbon backbone of rManNAc is numbered, and the active site residues of *N. meningitidis* are indicated. (**b**) Leg pathway. (**c**) Pse-pathway. For the Leg and Pse pathways, the C4 position of rManNAc (where there should be a second *N*-acetyl group) is indicated by an arrow. The C6 position, which does not carry a –OH group in Leg/Pse, is circled. Residues of cjNeuB2/cjNeuB3 that differ from that of nmNeuB are labeled. (**d**) Structure-sequence aligment of NeuB sequences from *Aliivibrio*, *Vibrio* and *Moritella viscosa* with nmNeuB. The sequence alignment is limited to the regions containing active site residues (numbered 1–12, circled in back with a gray background). Numbered residue positions correspond to the sequence of nmNeuB. The secondary structure information (nmNeuB) corresponds to the dssp data from its PDB entry. Residues are colored according to their properties as indicated in the legend. (**e**) mapping of sequence regions around the nmNeuB susbtrates. Regions represented several times contain several active site residues interacting at different location on the substrates.

Six of the eight targeted *M. viscosa* strains have two NeuB coding sequences identical to the ones from the 06/09/139 strain. The two remaining strains contain each one sequence, identical to either the Neu-pathway homolog (strain F57) or the Pse-pathway homolog (strain NVI-5482). This is consistent with the evolutionary relations between *M. viscosa* genomes according to which those two strains form their own clade^40^. It is also interesting to note that the strains with identical sequences were isolated from either salmond or rainbow trout, while the NVI-5482 and F57 were obtained from cod and lumpfish, respectively. For the *Aliivibrio* strains, two strains (*A. salmonicida* R8-68 and R8-70, isolated from the amphipod *Eurythenes gryllus*) have sequences identical two both homologs from LFI1238 (which was isolated from cod). The *A. logei* MR17-77 (from the sponge *Porifera indet*) and *A.* sp. R8-63 (from *Eurythenes gryllus*)) strains each have one hit that is almost identical (97% sequence identity for both) to the Leg-pathway homolog of *A. salmonicida* LFI1238 and *A. wodanis* (from salmon), respectively. While the clustering of aliivibrios does not reflect the type of host they were isolated from, it is in accordance with the phylogenetic data available for these strains^41, 42^. As expected, the NeuB sequence from *V. vulnificus* groups with that of *A. wodanis*, and is within the clade clustering Leg pathway sequences. The NeuB sequence from *Vibrio* sp. B9-25K2 is most similar (54% sequence identity) to the *M. viscosa* Pse-pathway homolog, although they do not belong to the same taxonomic family and were isolated from different organisms (sponge and salmon, respectively). This is expected since the only representative of the Pse pathway in our set of sequences is from *Moritellaceae*, and NeuB sequences are known to cluster according to pathway before species^28^. According to previously published phylogenetic data based on 16S rDNA sequences, this strain is closest to *V. anguillarum*, which does not produce NulOs^41^. Considering that phylogeny based on Multilocus Sequence Analysis (MLSA)^43, 44^ has been previously shown to be the preferable method for distinguishing between vibrios, we built a tree using this method with a subset of our target genomes (see Fig. 3). The tree only somewhat reflects the clades most recently proposed^44^ for the *Vibrionaceae*, which is most likely due to the small number of sequences considered in this study. The *M. viscosa* strains cluster together with some of the aliivibrios, which indicates that the set of genes chosen (*16SrDNA*, *ftsZ*, *gapA*, *gyrB*, *recA*, *rpoA* and *topA*) is not fully able to distinguish between the *Vibrionaceae* and *Moritellaceae* families. The tree does however provide a relevant phylogenetic context for *V.* sp. B9-25K2 for the scope of this study, so this was not investigated further.

#### Comparison of NeuB active site residues

The possibility of assigning NAB pathways solely based on the sequence of the NeuB homologs raises the question of what the determinants which discriminate between the pathways are. The genome of *Campylobacter jejuni* strains contain genes coding for NeuB variants for all three pathways (cjNeuB1-3), and was therefore chosen for studying the pathway-related characteristics of NeuB homologs^27^. The NCTC 11168 strain is able to produce Neu5Ac, Pse5Ac7Ac, and Leg5Ac7Ac, as well as derivatives of the Leg/Pse compounds (assuming that the acetamidino-derivatives are synthetized from the diacetylated compounds)^45^. Their sequences were modelled onto the structure of the NeuB homolog (Neu pathway) from *Nesseria meningitidis* (nmNeuB, PDB ID: 1XUU). The structure of their active sites was compared with its PEP-bound form (PDB ID: 1XUZ)^46^. The results, presented in Fig. 4 (panels a–d), show that while the active residues are strictly conserved between the Neu-pathway enzymes, they differ for the Leg- and Pse-pathway ones, as is expected. Indeed, the different pathways indicate that while NeuB from the Neu pathway acts on ManNAc for the production of Neu5Ac, the substrates leading to diacetylated Pse and Leg compounds are 2,4-diacetamido-2,4,6-trideoxyaltrose and 2,4-diacetamido-2,4,6-trideoxymannose, respectively^47–49^. Neither Leg nor Pse substrates carry a –OH group at C6 (circled in black in panels b and c), which explains the variations observed for the residues neighboring it. It is interesting to note that the OH group carried by the Neu substrate in C4 is interacting with a water molecule, which is probably not present in the cjNeuB2 (Leg) and cjNeuB3 (Pse) active sites. Both Leg and Pse have an *N*-acetyl group at this position, and the water molecule could be compensating for the *N*-acetyl group. In addition to carrying a different substituent, C4 also has a different configuration in the Leg (S) and Pse (R) substrates (same orientation as the –OH of rManNAc for the Leg substrate). This could account for the variability of residues in proximity to the C4 position (indicated by a black arrow in panels b and c). Most interesting is the F129 residue of cjNeuB3 clashing with the *N*-acetyl group carried in C2 by the Neu substrate. This group has a different orientation in the Pse substrate (C2 has a S configuration) compared to the others. The conserved part of the active site, around the PEP substrate and the C1 of rManNAc, is in agreement with the proposed mechanism of NeuB enzymes, which involves this particular region^46^. This area is conserved in all three isozymes, while the area binding the rest of the sugar molecule is less so, allowing for specificity at C2 via position 132 (F129 in NeuB3) and at C4-C6 via the regions corresponding to the loop S2 and helix H4 of nmNeuB (positions 70–85).

Our set of unique NeuB sequences was also used in a sequence-structure alignment, and the active site residues can be described as 12 regions on the NeuB sequences (see Fig. 4 panel d; residues indicated by their nmNeuB sequence number). Those regions were mapped around the nmNeuB substrate structure in Fig. 4 (panel e) as a simpler way to refer to active site sublocations and their corresponding residues. The multiple alignment strengthens the observations made by structure comparison, with the regions surrounding the sugar chain (from C2) less conserved than the others. Regions 5, 6 and 8 each contains several active site residues interacting with the substrates at different locations. PEP is surrounded by positions 110 (region 5); 129, 131 and 132 (region 6); 182 and 184 (region 8), with positions 132, 182 and 184 less conserved. Position 132 corresponds to the aforementioned G132/F129 residues of cjNeuB1/cjNeuB3, which may influence specificity at C2. The multiple alignments confirms that the Gly appears to be conserved within the Neu and Leg pathways, while the Phe is conserved for the Pse pathway. A rapid check with a larger set of sequences from public databases shows that the positions are mostly but not strictly conserved. Position 184 is occupied by either Asn or Tyr in the Neu pathway, while the Leg and Pse pathways have at this position a conserved Tyr and Ser, respectively. In the nmNeuB structure, N184 interacts at least weakly with all susbtrates. Whether the differences in residue between pathways is a matter of susbtrate specificity is not clear. The same can be said of position 182, where the differences seem more to reflect phylogenetic distance than function. Amongst the Neu-pathway homologs, the sequence from *A. salmonicida* LFI1238 is more similar to that of the Leg pathway homologs, at least in part. This homolog is thought to accept 4-acetylated ManNAc (ManNAc2) as a natural substrate rather than the monoacetylated form, even though it is capable of utilizing it^34^. Considering that there may be some flexibility near C4 with the water molecule and that ManNAc2 is identical to LegAc2 at this position, the preference for ManNAc2 is supported by the sequence-structure comparison. Amongst the Leg pathway homologs, the closely related homologs from *A.* sp. R8-63 and *A. wodanis* share all their active site residues, as do the *A. salmonicida* LFI1238 and *A. logei* MR17-77 strains. The two groups differ from each other in regions 3-8, indicating that they may have a different susbstrate specificity. 8eLeg5Am7Ac was detected in *A. salmonicida* LFI1238, while *A. wodanis*, whose cluster is closest to that of *Vibrio vulnificus* CMCP6, might produce Leg5Ac7AcAla (or a variant of it)^34, 39^. The corresponding biosynthesis pathways seem to involve different NeuB susbtrates, which is consistent with the observations made here.

**Figure 5:**
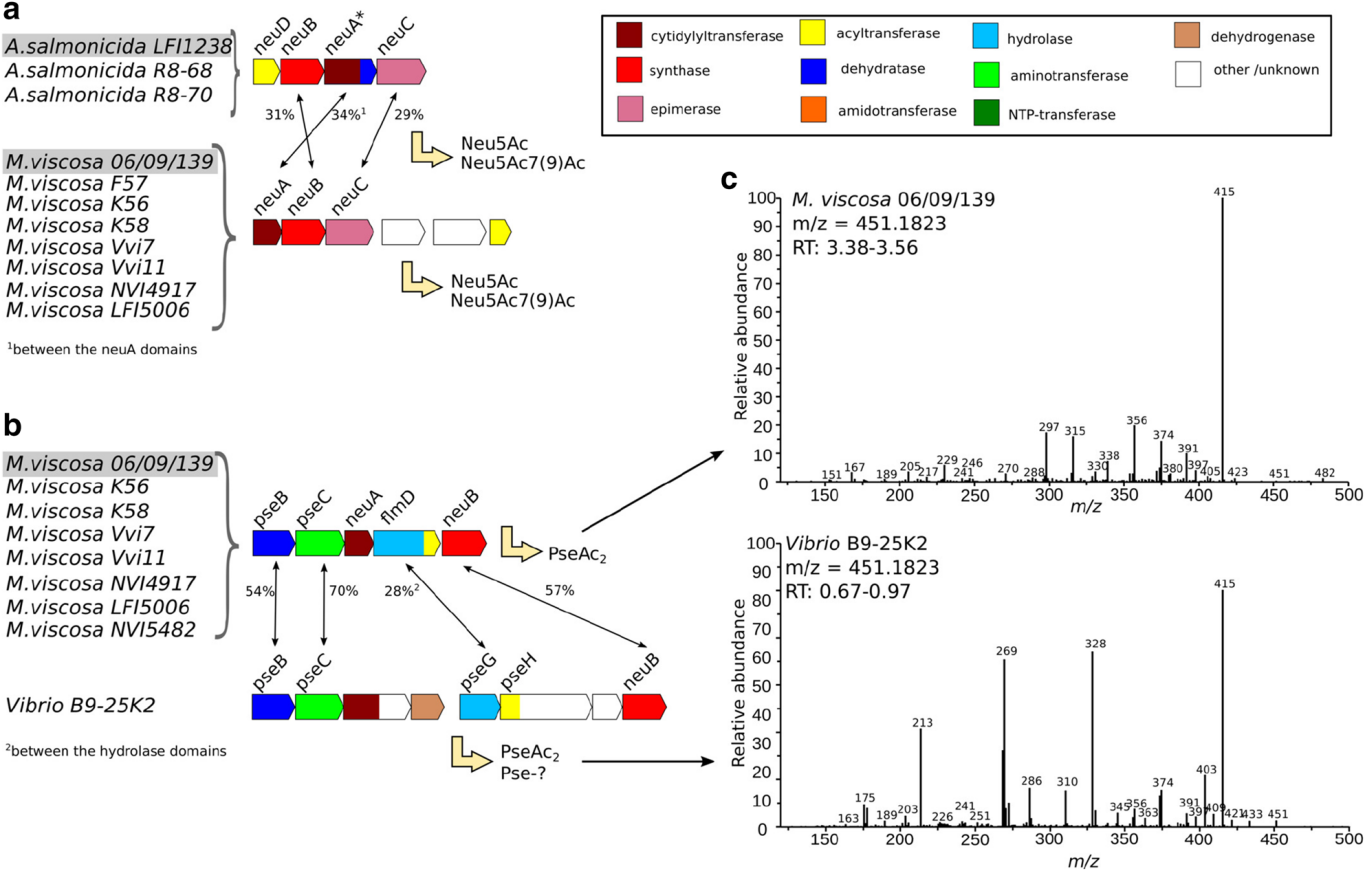
Unique Neu and Pse *nab* clusters amongst *Aliivibrio*, *Vibrio*, and *Moritella viscosa* strains. (**a**, **b**) The translated nucleotide sequence of each gene of each cluster was compared globally to the protein sequences coded by the *nab* clusters of *A. salmonicida* LFI1238, *A. wodanis* 06/09139, and *M. viscosa* 06/09/139. Each gene is colored according to the function of the encoded protein, as stated in the figure legend. Homologs are indicated by black arrows, with the percentage of sequence identity next to it. Predicted (previously identified in the case of Neu clusters) products are indicated. (**c**) MS/MS spectra of the quinoxalines corresponding to PseAc2 (*Moritella viscosa* and *Vibrio*). Top: ITMS + cESI with full MS/MS spectra of mass 451.1823 at time 3.38–3.56 min (*Moritella viscosa*). Bottom: ITMS + cESI with full MS/MS spectra of mass 451.1823 at time 0.67–1.06 min (*Vibrio* sp. B9-25K2).

**Figure 6:**
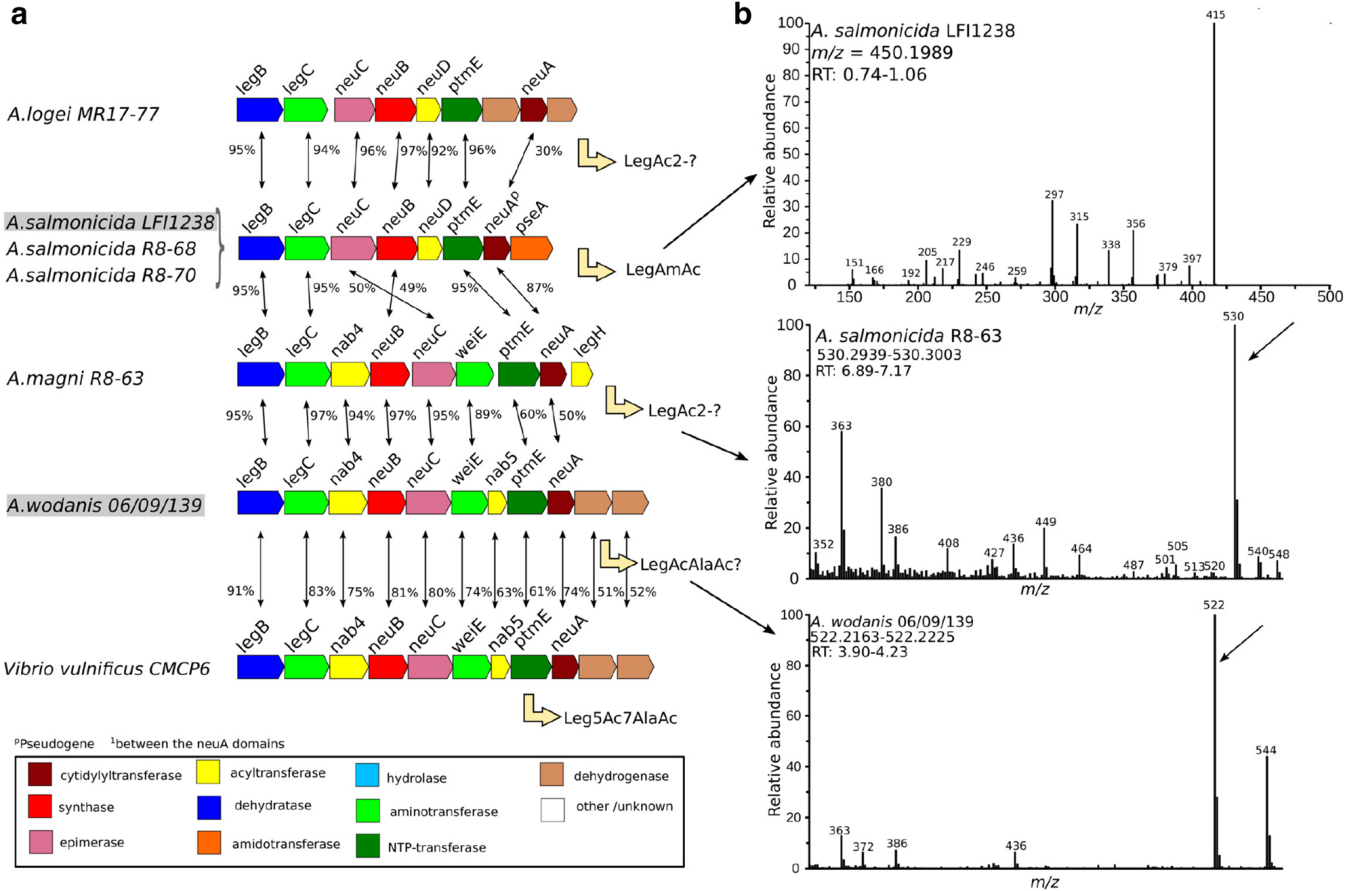
Unique Leg *nab* clusters amongst *Aliivibrio* strains. (**a**) The translated nucleotide sequence of each gene of each cluster was compared globally to the protein sequences coded by the *nab* clusters of *A. salmonicida* LFI1238, *A. wodanis* 06/09139, and *M. viscosa* 06/09/139. Each gene is colored according to the function of the encoded protein, as stated in the figure legend. The percentage of sequence identity between homologs is indicated by black arrows, with the value next to it. (**b**) MS and MS/MS spectra of the quinoxalines corresponding to Leg compounds. From top to bottom: ITMS + cESI with full MS/MS spectra of mass 450.1989 at time 0.74–1.06 min (*A. salmonicida* LFI1238); FTMS + pESI full MS spectra at $$m/z= $$ (*A. salmonicida* R8-68); FTMS + pESI full MS spectra at $$m/z= $$ (*A. wodanis*).

**Figure 7:**
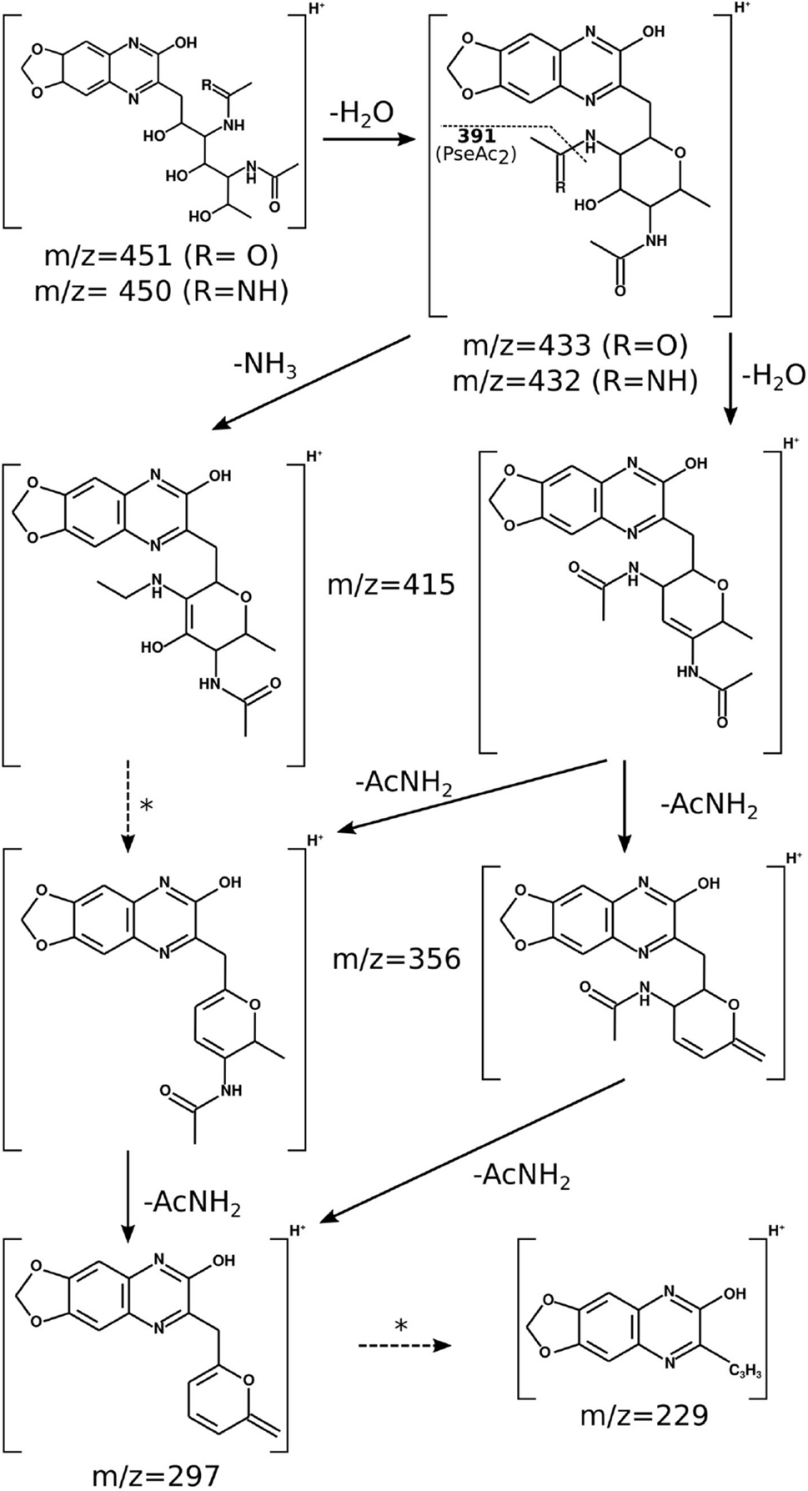
Proposed fragmentation route of LegAmAc (*A. salmonicida*) and PseAc2 (*M. viscosa*). The major peaks of the MS2 spectra (ITMS + cESI with full MS2) for both compounds were assigned to fragments of corresponding mass, according to the fragmentation route of Neu quinoxalines described by Klein *et al.*^51^. Multiple steps are marked with dashed arrows and asterisks (*).

### *nab* cluster identification and analysis

The genome regions surrounding each NeuB homolog hit were investigated for the presence of putative *nab* clusters. The results, presented in Figs. 5 (panels a and b) and 6 (panel a), reveal that hits (or queries) with identical NeuB sequences had identical clusters (identical gene composition, sequences and organization). This is especially interesting in the case of the Leg pathway cluster from *A. salmonicida* LFI1238 (see Fig. 6, panel a), where the pseudogene sequence for NeuA is conserved with that of *A. salmonicida* R8-68 and R8-70. Furthermore, where the NeuB sequences are not identical, the clusters are different, even if the sequences are highly similar. Indeed, the Leg clusters from *A. logei* MR17-77 and *A.* sp. R8-63, for which the NeuB sequences share 97% identity with that of *A. salmonicida* LFI1238 and *A. wodanis*, respectively, have a different architecture composed of different genes. When homologs are present, their sequences are similar, but not identical. It is worth noting that the Leg cluster of *A.* sp. R8-63 is closest to that of *A. wodanis* for its first part (up to the second aminotransferase), but then shows more similarity with that of *A. salmonicida* LFI1238 (PtmE and NeuA sequences).

In order to confirm if this trend is observed for other species, we investigated the set of NeuB sequences similar to that of *Vibrio* B9-25K2, leading to the conclusion that when NeuB sequences are identical, the corresponding clusters might share a similar architecture with the same number of proteins coding for the same homologs in the same order, but they are not necessarily all identical in protein sequence (data not shown).

The cluster of *Vibrio* B9-25K2, for which the NeuB sequence was most similar to that of Pse pathway homolog from *M. viscosa*, contains homologs of sequences coding for PseB, PseC and PseG, which confirms its appartenance to this pathway (see Fig. 5, panel b). It also contains a homolog of PseH as a domain in a bi-functional protein for which the second domain is of unknown function, but that is associated to lipid metabolism. A conserved domains analysis of the cluster coding sequences revealed that the bi-functional enzyme encoded after PseC contains a putative cytidylyltransferase domain similar to the SpsF spore coat protein, indicating that it may be replacing the NeuA homolog absent from this cluster. The other coded domain is that of an aldo-keto reductase. Following this gene is a sequence coding for a dehydrogenase. The PseH homolog is followed by a gene coding for a hypothetical protein, with a domain similar to that of methylmalonyl CoA epimerase. The clusters corresponding to the closest NeuB sequences from public databases also contain the hypothetical protein and the bi-functional PseH homolog, both with around 60% sequence identity compared to that of *Vibrio* B9-25K2 (data not shown). A likewise search for the bi-functional cytidylyltransferase yielded a different set of strains sharing similar architectures, but none for which the NulO content has been investigated (data not shown).

From the analysis of the NeuB homologs, we were able to hypothetize that the NeuBs from *A. logei* MR17-77 and R8-63 most likely produces Leg compounds, with the one from MR17-77 producing the same as that of *A. salmonicida* LFI1238 (LegAc2) and R8-63 the same as that of *A. wodanis* (Leg5Ac7AcAla or similar, since the sugar in *A. wodanis* has not been identified yet). However, the composition of their clusters is different (see Fig. 6, panel a), and they most likely ultimately each produce different compounds. The NeuB from *Vibrio* B9-25K2 seems to produce a Pse related NulO, but the lack of close sequences with identified NulO content in the corresponding organisms prevented a more precise hypothesis. The results from the cluster analysis are in agreement with these suggestions, and they suggest that *Vibrio* B9-25K2 may produce a diacetylated Pse on account of the presence of a PseH homolog (see Fig. 5, panel b). The presence of enzymes of unknown function might indicate further modifications of produced compound. The NulO content for the Neu pathway clusters has been previously identified, with both *A. salmonicida* LFI1238 and *M. viscosa* producing Neu5Ac and Neu5Ac7(9)Ac^32–34, 38^.

### Analysis of bacterial NulO content by LC-MS and MS/MS

The NulO content of *A. salmonicida* LFI1238, *A.* sp. R8-63, *M. viscosa*, *A. wodanis* and *Vibrio* sp. B9-25K2 was roughly investigated by LC-MS, as a way to rapidly check the NulO predictions^50, 51^. In addition to this, MS/MS was used to investigate the presence of LegAmAc and Leg/PseAc2 in *A. salmonicida* LFI1238, *M. viscosa* and *Vibrio* sp. B9-25K2. Both analyzes were performed on DMB-labelled samples, with NulOs present as quinoxalines (if any). The results are summarized in Figs. 5 (panel c) and 6 (panel b), with the spectra next to their corresponding clusters. Detailed results are available as Supplementary Information (see Figures S2 and S3). The quinoxalines corresponding to the previously identified NeuAc (NeuAcQ: m/z = 426), NeuAc2 (NeuAc2Q: m/z = 468) and LegAmAc (LegAmAcQ: m/z = 450) of *A. salmonicida* LFI1238 and *M. viscosa* were detected as expected (see supplementary materials)^34, 38, 51^. MS/MS had not been done yet for LegAmAcQ (*m*/*z*[M+H]+ = 450.19887323), and is presented in Fig. 6 (panel b, top). The Leg5Ac7AcAla compound was not identified at the time of the experiment, and masses related to its quinoxaline were not investigated^39^. The presence of quinoxalines corresponding to PseAc2 (PseAc2Q: m/z = 451) was detected in both *M. viscosa* and *Vibrio* B9-25K2 (see Supplementary Information). Interestingly, they had different retention times, and their MS/MS spectra (PseAc2Q: *m*/*z*[M+H]+ = 451.1823402) reveal different fragmentation patterns (see Fig. 5, panel c). Instead, it is the spectra for LegAmAcQ (*A. salmonicida* LFI1238) and the LegAmAcQ from *M. viscosa* that are similar. The samples from *A.* sp. R8-63 and *A. wodanis* had peaks at m/z= 530 and 522, respectively (see Fig. 6, panel b). While the former does not correspond to any identified NulO by this method, the latter can be tentatively assigned to the Leg5Ac7AcAlaQ compound observed in *Vibrio vulnificus*^39^.

As mentioned above, the MS/MS spectra of the PseAc2 compounds from *M. viscosa* and *Vibrio* B9-25K2 show separate fragmentation patterns (see Fig. 5, panel c). This suggests that either the *N*-acetyl groups are substituents to different carbons of the Pse backbone, or that some of the asymmetrical carbons have a different geometry. In the latter case, the strains would produce epimers of PseAc2. No reports of a di-*N*-acetylated NulO carrying groups at positions other than C5 and C7 exist yet, and the only pathway known to produce epimers of its NulO is the Leg pathway^11–14^. The determination of the absolute configuration of PseAc2 from *Vibrio* B9-25K2, as well as studies targeting the proteins of its cluster should provide the necessary information. A common feature of all the presented MS/MS spectra is that the parent ions are either weak or absent, as is expected from molecules rich in alcohol groups (see Fig. 5, panel c) for the PseAc2 spectra and Fig. 6 (panel b, top) for the LegAmAc spectrum). The base peak, at m/z = 415, can result from the loss of either two water molecules from the PseAc2 compounds or one water and one ammonia from LegAmAc. The fragmentation route proposed by Klein et al., which involves the formation of a ring between the C4 and C8 of the NulO moiety, is consistent with the observed peaks (see Fig. 7). Indeed, the ring formation corresponds to the loss of a water molecule gives a compound at $m/z= 433$ for PseAc2 (432 for LegAmAc), where a small peak is observed for *Vibrio* B9-25K2. This route leads, when the ring substituents are removed in C5-C7, to a compound with $m/z= 297$ which loses the ring to form the compound at $m/z = 229$. The compound from *Vibrio* B9-25K2 most likely forms another ring, due to a different proximity of the groups either because it is an epimer of Pse5Ac7Ac, and/or because the *N*-acetyl groups are carried by other positions.

This preliminary experimental characterization strengthens the hypotheses made after analysis of the NeuB homologs and the nab clusters concerning the type of sugar produced by each cluster. A similar approach has already been used successfully in several species and the method appears robust, although it is limited to detecting compounds similar (by either mass or gene cluster) to previously identified sugars^24, 39, 52^. Obviously, a more thorough experimental analysis is needed to fully characterize the compounds detected. The results should thus be treated with caution.

## Conclusion

This study investigated the *nab* clusters from a set of bacteria from the *Vibrionaceae* and *Moritellaceae* families, based on the sequence comparison of their NeuB homologs. We found that each unique NeuB sequence corresponded to a unique *nab* cluster, each potentially producing different NulOs. This opens the possibility of screening sequence databases as the first step in the identification of previously undescribed NulOs. It also allows for the mapping of potential NulO diversity within species with published NeuB sequences and/or genomes. Considering that NeuB sequences cluster according to NAB pathway rather than species, we used sequence-structure comparison to identify putative substrate specificity regions for the NeuB homologs. In particular, we located a critical position occupied by either a glycine (Neu and Leg pathways) or a phenylalanine (Pse pathway) which seems to discriminates between NAc orientation at the C2 position of NeuB substrates. Further study of NeuB specificity not only opens for a better prediction of its substrates and products, but also the tailoring of already characterized enzymes for the production of various NulOs.

Going further, the study of the nab clusters associated with NeuB sequences of interest allows for better hypotheses concerning NulO structure as well as the detection of new NAB proteins previously unidentified, such as the non-NeuA homolog cytidylyltransferase from *Vibrio* B9-25K2. Preliminary results from mass spectrometry analysis of the NulO content of the target organisms both confirmed the presence of predicted sugars, but also revealed a new compound in *A.* sp. R8-63 for which the corresponding quinoxaline has a *m*/*z* of 530, which awaits identification.

Taken together, the results support the use of NeuB sequence comparison as the basis for screening genomic data of NulO producing organisms, with the purpose of finding new protein and/or monosaccharide targets. In the case of *Vibrio* B9-25K2, which seems to produce a variant of PseAc2 with a different configuration and which cluster presents several unknown coding sequences, further studies might uncover a new branch of the Pse pathway.

## Methods

### Strains and sequences

The source information for the genomes used in this study is summarized in Table 1.

### Sequence alignments and phylogentic analyses

The target genomes were, if necessary, annotated using the GePan pipeline of the Galaxy server at the University of Tromsø^53, 54^. Glimmer v3.2 was used for gene prediction and BLASTp for database search within Bacteria^55, 56^.

The protein sequences for the NeuB homologs from *A. salmonicida* LFI1238 (accession numbers WP_012551408.1 and WP_012549051.1), *M. viscosa* 06/09/139 (WP_045111757.1 and WP_045111735.1), and *A. wodanis* 06/09/139 (WP_045100955.1) were used as query for a similarity search against the set of target genomes using the BLAST suite^55^. The NeuB sequence from *V. vulnificus* CMCP6 was added to the set and unique NeuB protein sequences were thereafter aligned with MUSCLE, using default settings^57, 58^.

The evolutionary history of the NeuB sequences was inferred using the Neighbor-Joining method^59^, using the NeuB sequence from *Legionella moravica* (WP_028385079.1) as outgroup. The final dataset was composed of 10 sequences and 318 positions, with all positions containing gaps and missing data eliminated (complete deletion option). The evolutionary distances were computed using the Poisson correction method^60^ and are in the units of the number of amino acid substitutions per site. The rate variation among sites was modeled with a gamma distribution (shape parameter = 5). Evolutionary analyses were conducted in MEGA X^61^.

The evolutionary history of *Vibrio* sp. B9-25K2 was inferred using Multilocus Sequence Analysis (MSLA)^43^. The sequences for the 16SrDNA, *ftsZ*, *gapA*, *gyrB*, *mreB*, *pyrH*, *recA*, *rpoA* and *topA* genes were retrieved for *V.* sp. B9-25K2, *A. salmonicida* LFI1238, *A. logei* MR17-77, *A.* sp. R8-63, *A. wodanis* 06/09/139, *V. vulnificus* CMCP6, *V. anguillarum* NB10, *M. viscosa* 06/09/139, *M. viscosa* LFI5006 and *Legionella moravica* DSM19234 (outgroup). The accession numbers and nucleotide positions are provided in Supplementary Information (Table S1). Each gene group was aligned in MUSCLE and the sequences from the same strains were concatenated (in the same order for all). The tree was calculated using the Neighbor-Joining method^59^ and tested with bootstrap (1000 replicates). The evolutionary distances were computed using the Jukes–Cantor method^62^ and are in the units of the number of base substitutions per site. The rate variation among sites was modeled with a gamma distribution (shape parameter = 5). This analysis involved 10 nucleotide sequences. All positions containing gaps and missing data were eliminated (complete deletion option). There were a total of 8050 positions in the final dataset. Evolutionary analyses were conducted in MEGA X^61^.

### *nab* cluster identification and analysis

The positive NeuB hits were located on the target genomes and the surrounding area was investigated for the presence of putative *nab* gene clusters, using the Artemis software for viewing^63^. The cluster regions were determined by the gene content and direction of the relevant areas. Gene names were assigned according to protein sequence similarity to known *nab* genes, using the most comprehensive (to the authors) denominations.

Identified clusters were analyzed for the function of each coding sequence composing them by performing sequence similarity searches within the non-redundant protein sequence database (BLAST) as well as domain searches within the Conserved domain Database^64^.

### Homology modelling of *C. jejuni* NeuB homologs and active site comparison

The sequences for the NeuB homologs of *C. jejuni* NCTC11168 (WP_002858213.1, WP_002864265.1, and WP_002870258.1) were retrieved from public databases and used as targets for homology modelling using the NeuB homolog from *N. meningitidis* (nmNeuB, PDB IDs: 1XUU, 1XUZ) as a template^46, 65^. The modelling was performed on the SWISS-MODEL server^66^. The unique NeuB sequences from the target strains and the aforementioned *C. jejuni* sequences were also aligned to nmNeuB using the PROMALS3D server (https://doi.org/10.1093/nar/gkn072). Sequence regions with active site residues were located by measuring distances to the substrates in the structures as well as using the PDBePISA server for nmNeuB (https://doi.org/10.1016/j.jmb.2007.05.022).

### Nonulosonic content release and derivatization

Cultures (15 mL) of *A. salmonicida* LFI1238, *M. viscosa* 06/09/139, and *Vibrio* B9-25K2 were grown in liquid LB media containing 2.5% NaCl (48 h, 12 °C, 200 rpm). 3 mL pellets were harvested and washed with dH$_2$O before they were resuspended in 0.1 $\upmu$L phenylmethane sulfonyl fluoride (PMSF). After a 15 min incubation on ice, 200 $\upmu$L acetic acid (2M) and 2 $\upmu$L butylated hydroxytoluene (BHT, 1%) was added. The samples were thereafter incubated for 3 h at 80 $^\circ$C and spun down for 10 min at 13,000 rpm. The supernatant was collected and filtered (Amicon 10K spin column) in order to remove large molecules. The filtrate was dried for 2 h using a speed-vac, and the resulting samples were stored at − 20$^\circ$C until use.

Samples were resuspended in 10 $\upmu$L $\hbox {dH}_{2}$O before performing the labelling reaction with 1,2-diamino-4,5-methylenedioxybenzene (DMB, from TaKaRa) according to the manufacturer’s instructions. The reaction mixtures were incubated in the dark at 50 $^\circ$C for 2.5 h.

### Mass spectrometry analyses

The quinoxaline (Q) content of the samples described in the previous section was analyzed by HPLC-MS/MS using the procedure described by Gurung et al.^34^. Water with 0.1% formic acid (A) and acetonitrile with 0.1% formic acid (B) were used for the HPLC-MS elution gradient (see a previous file and put the gradient here), at a flow rate of 400 $\upmu$L/min. Tandem mass spectrometry was performed on samples containing compounds corresponding to masses equivalent to that of LegAmAcQ (*m*/*z*[M+H]+ = 450.19887323) and LegAc2Q/ PseAc2Q (*m*/*z*[M+H]+ = 451.1823402). The scan range was *m*/*z* 350–550.

### Graphical output generation

Gene clusters were rendered in SVG graphics using scripts written by the author. The scripts are available through the python-bioinformatics repository on GitHub^67^. Molecular structures were obtained from PubChem and modified in Molview and Pymol^68, 69^. All figures were prepared using Inkscape^70^.

## Electronic supplementary material

Supplementary Information 1.
