# Supplementary material for: Sequence analysis of nonulosonic acid biosynthetic gene clusters in Vibrionaceae and Moritella viscosa
Source: Sci Rep. 2020 Jul 20;10:11995. doi: 10.1038/s41598-020-68492-3 (PMC7371886; doi:10.1038/s41598-020-68492-3)
Supplement: Supplementary file 1 — Supplementary Information 1. [file 41598_2020_68492_MOESM1_ESM.pdf]

Supplementary Information

Sequence analysis of nonulosonic acid biosynthetic gene clusters in *Vibrionaceae* and *Moritella viscosa*

Marie-Josée Haglund Halsør[1], Bjørn Altermark[1], and Inger Lin Uttakleiv Ræder[1,*]

[1]The Norwegian Structural Biology Centre (NorStruct), Department of Chemistry, UiT- The Arctic University of Norway, N-9037 Tromsø, Norway
[*]inger.l.rader@uit.no

Table S1. Sequences used for Vibrio sp. B9-25K2 phylogeny.

	Accession and ranges		
Strain *reference*	16SrDNA	*ftsZ*	*gapA*
Aliivibrio logei MR17-77[1]	1434910-1436398	3523605-3524840	2928874-2929790
A. wodanis 06/09/139[1]	LN554846.1 (506389-507875)	464002-465231	1057819-1058735
A. sp. R8-63[1]	2991032-2992518	80694-81932	4041528-4042444
A. salmonicida LFI1238[2]	NC_011312.1 (87128-88616)	2857507-2856272	1984565-1985481
Moritella viscosa 06/09139[3]	LN554852.1 (31135-32623)	4800155-4798974	3844953-3845966
M. viscosa LFI 5006[3]	FPLG[a] (81[b]; 98-1586)	88[b]; 80018-81199	53[b]; 23338-24351
Vibrio anguillarum NB10[4,5]	LK021130.1 (233633-235121)	2515501-2516718	1112491-1113410
V. sp. B9-25K2[1]	3642484-3643981	242232-243211	4201830-4202749
V. vulnificus CMCP6[6,7]	AE016795.3 (475743-477229)	588232-587018	3210958-3211877
	gyrB	*mreB*	*pyrH*
Aliivibrio logei MR17-77[1]	3216464-3217646	169476-170505	3021801-3022387
A. wodanis 06/09/139[1]	LN554846.1 (11401-12583)	2635881-2634838	2379026-2379612
A. sp. R8-63[1]	465716-466898	2026880-2027922	1570486-1571072
A. salmonicida LFI1238[2]	NC_011312.1 (11398-12580)	541014-542057	2595577-2596154
Moritella viscosa 06/09139[3]	LN554852.1 (5072328-5073736)	4853906-4854946	4396130-4396716
M. viscosa LFI 5006[3]	FPLG[a](94[b]; 26583-27765)	88[b];134953-135993	05[b];39318-39889
Vibrio anguillarum NB10[4,5]	LK021130.1 (11313-12710)	357047-358090	2368060-2368788
V. sp. B9-25K2[1]	5040401-5041803	37279-38250	3344816-3345402
V. vulnificus CMCP6[6,7]	AE016795.3 (999953-1001349)	1432966-1434009	1856011-1856597
	recA	*rpoA*	*topA*
Aliivibrio logei MR17-77[1]	4960-6003	3796758-3797685	2769318-2771957
A. wodanis 06/09/139[1]	LN554846.1 (616746-617792)	2765320-2766247	1218882-1221515
A. sp. R8-63[1]	1850404-1851453	2759195-2760122	2965401-2968040
A. salmonicida LFI1238[2]	NC_011312.1 (702343-703386)	375824-376751	1266401-1269040
Moritella viscosa 06/09139[3]	LN554852.1 (529995-531035)	4908357-4907374	1552072-1554717
M. viscosa LFI 5006[3]	FPLG[a](05[b]; 60952-61992)	90[b]; 852-1835	33[b];82187-84832
Vibrio anguillarum NB10[4,5]	LK021130.1 (529112-529908)	2696727-2697657	1281671-1284301
V. sp. B9-25K2[1]	255724-256681	4035345-4036275	4894973-4897596
V. vulnificus CMCP6[6,7]	AE016795.3 (1563785-1564703)	752353-753283	2129301-2131925

[a] Whole Genome Sequencing (WGS) project identifier
[b] Contig number

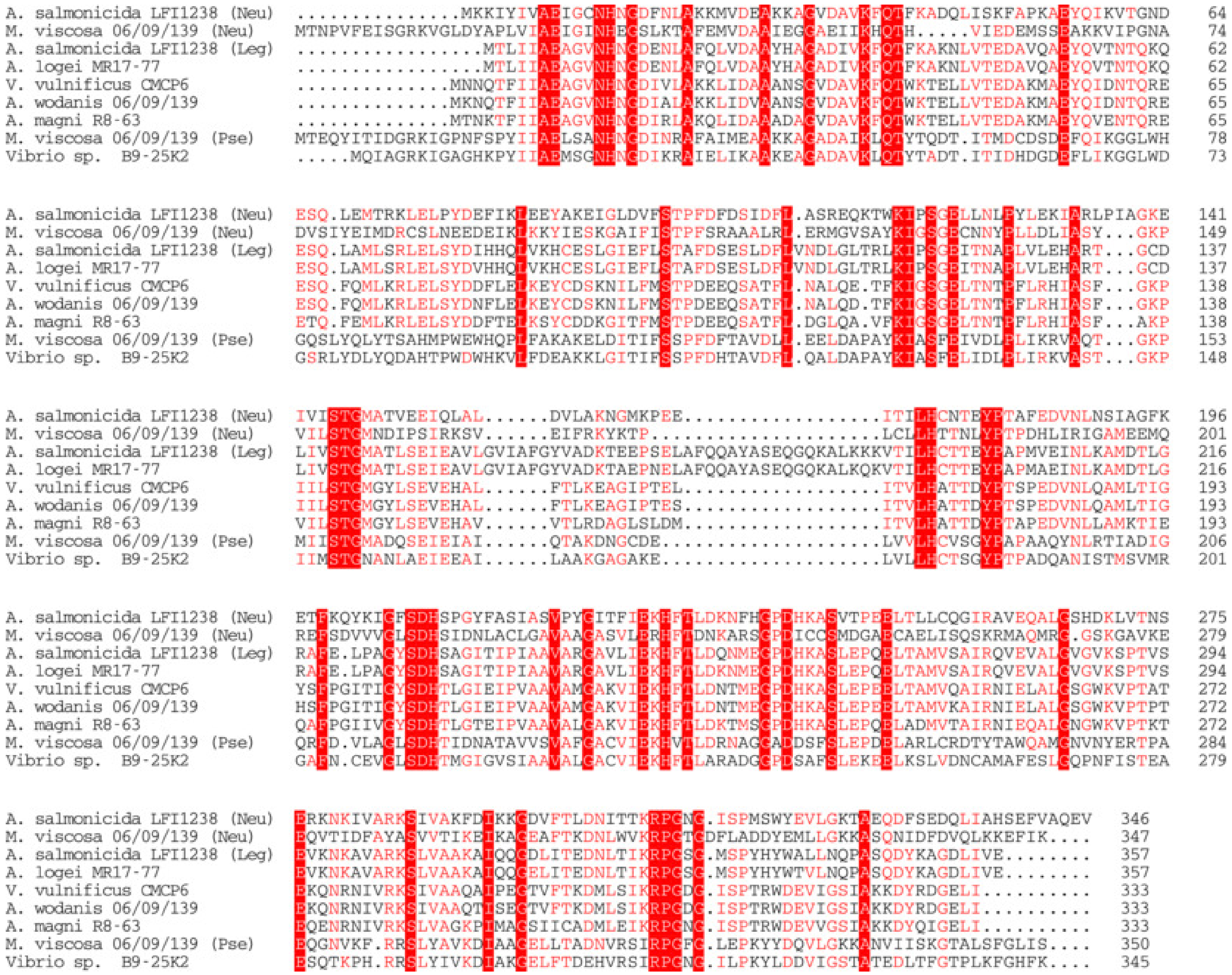

Figure S1. Multiple alignment of unique NeuB sequences. The sequences from *A. salmonicida* LFI1238 (WP_012551408.1 and WP_012549051.1), *A. magni* R8-63 and *A. logei* MR17-77 were aligned with that of *M. viscosa* 06/09/139 (WP_045111757.1 and WP_045111735.1) and *A. wodanis* 06/09/139 (WP_045100955.1) as well as *Vibrio* B9-25K2. The alignment was done using MUSCLE[8,9].

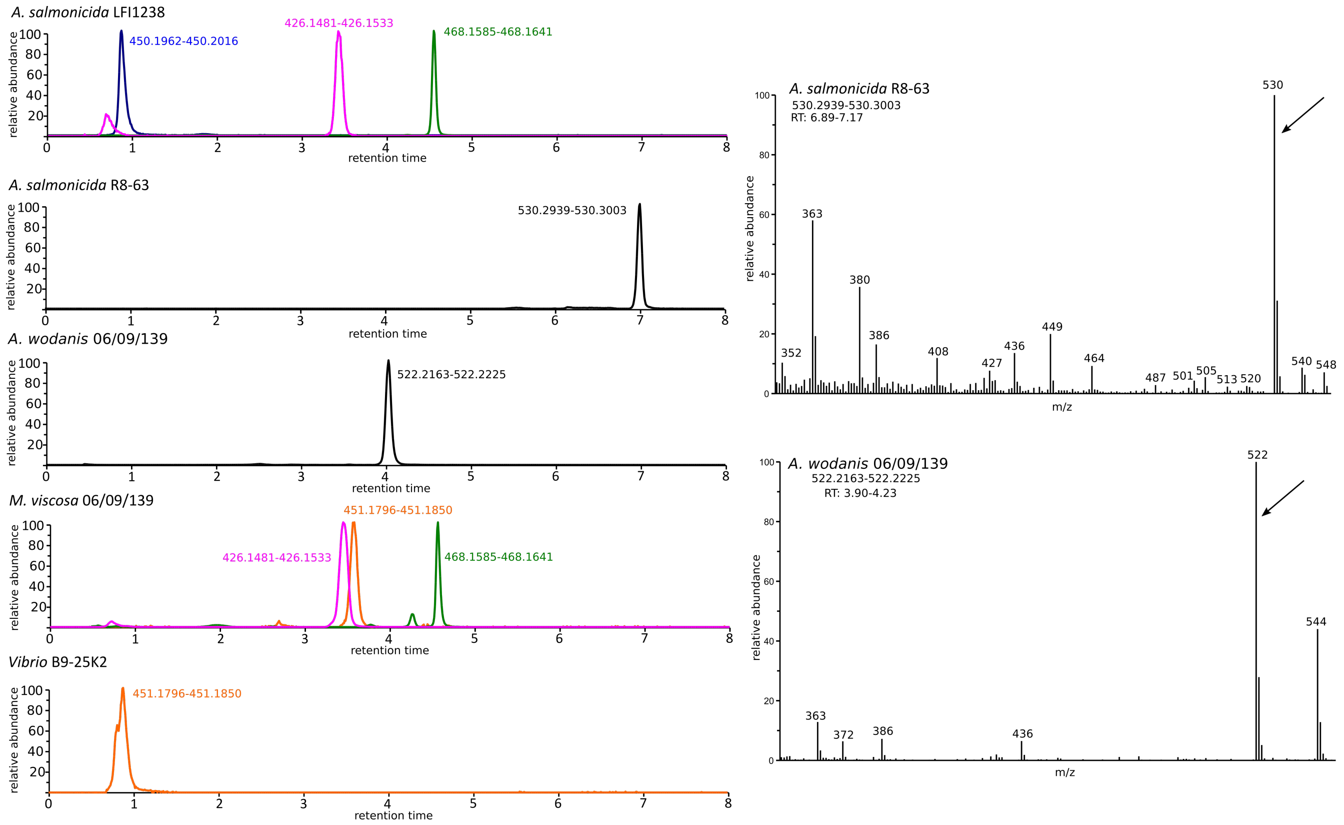

Figure S2. LC-MS analysis of bacterial NulO content derivatized with DMB. Left: FTMS+ pESI full MS spectra. The spectra are represented for each species as a function of relative abundance versus retention time (in minutes). When several mass ranges are considered, the spectra are combined in a single graph using different colors for each curve (blue, pink, green, and orange). The mass range (m/z) corresponding to each peak is indicated next to it. Right: Mass spectra for the peaks from *A. salmonicida R8-63* and *A. wodanis*.

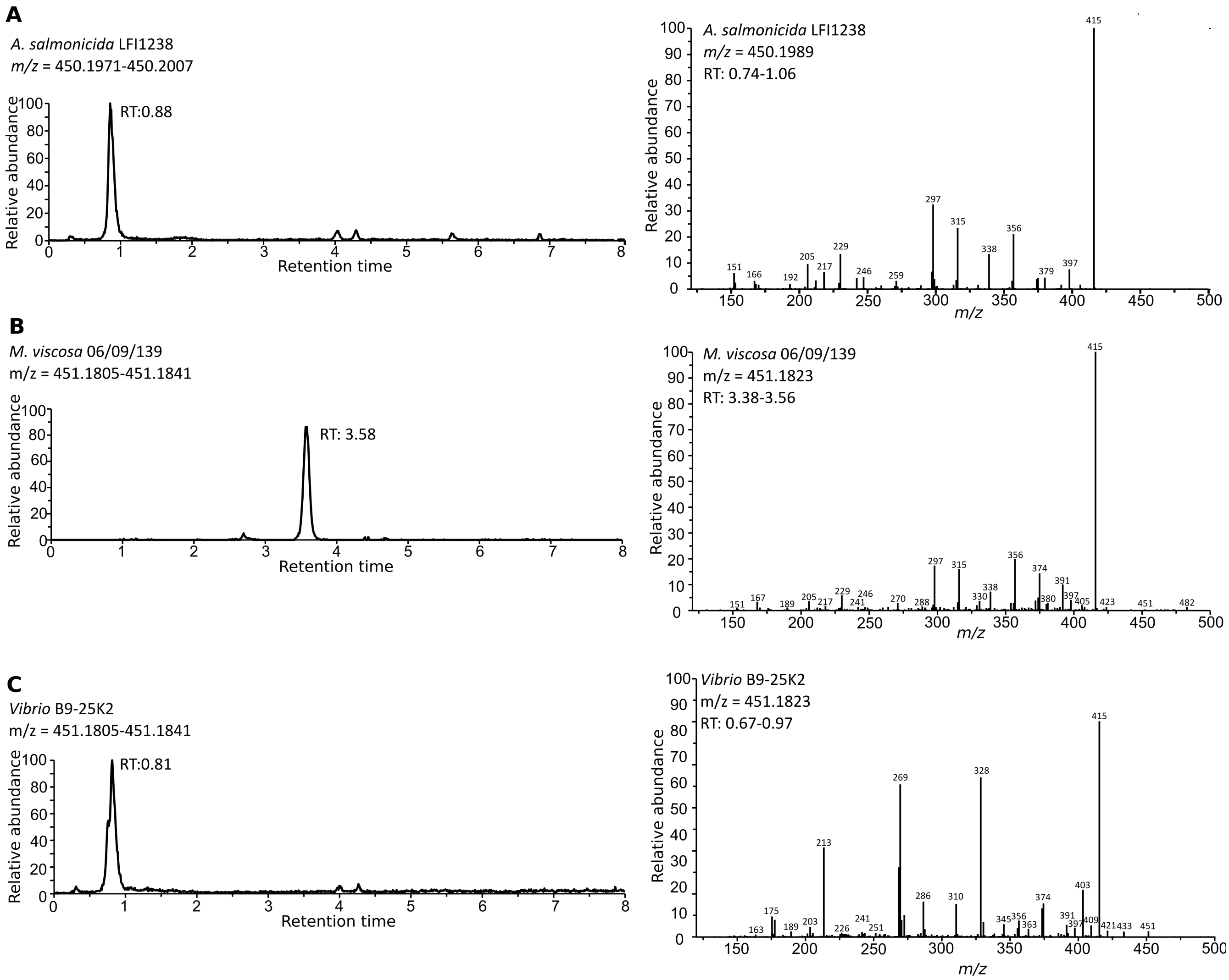

Figure S3. Mass spectrometry analysis of putative LegAcAm and PseAc2 compounds. A. Analysis for *A. salmonicida* LFI1238. Left: FTMS+ pESI full MS spectra at $m/z = 450.1971 - 450.2007$. Right: ITMS + cESI with full MS2 spectra of mass 450.1989 at time 0.74-1.06 minutes. B. Analysis for *M. viscosa* 06/09/139. Left: FTMS+ pESI full MS spectra at $m/z = 451.1805 - 451.1841$. Right: ITMS + cESI with full MS2 spectra of mass 451.1823 at time 3.38-3.56 minutes. C. Analysis for Vibrio B9-25K2. Left: FTMS+ pESI full MS spectra at $m/z = 451.1805 - 451.1841$. Right: ITMS + cESI with full MS2 spectra of mass 451.1823 at time 0.67-1.06 minutes.

References

1. Purohit, A. A. *et al.* Presence of acyl-homoserine lactones in 57 members of the *Vibrionaceae* family. *J. Appl. Microbiol.* **115**, 835–847, DOI: https://doi.org/10.1111/jam.12264 (2013).

2. Hjerde, E. *et al.* The genome sequence of the fish pathogen *Aliivibrio salmonicida* strain LFI1238 shows extensive evidence of gene decay. *BMC Genomics* **9**, DOI: https://doi.org/10.1186/1471-2164-9-616 (2008).

3. Karlsen, C., Hjerde, E., Klemetsen, T. & Willassen, N. P. Pan genome and CRISPR analyses of the bacterial fish pathogen *Moritella viscosa*. *BMC genomics* **18**, 313, DOI: https://doi.org/10.1186/s12864-017-3693-7 (2017).

4. Eiler, A. & Bertilsson, S. Detection and quantification of *Vibrio* populations using denaturant gradient gel electrophoresis. *J. Microbiol. Methods* **67**, 339–348, DOI: https://doi.org/10.1016/j.mimet.2006.04.002 (2006).

5. Holm, K. O., Nilsson, K., Hjerde, E., Willassen, N. P. & Milton, D. L. Complete genome sequence of *Vibrio anguillarum* strain NB10, a virulent isolate from the Gulf of Bothnia. *Standards Genomic Sci.* **10**, DOI: https://doi.org/10.1186/s40793-015-0060-7 (2015).

6. Kim, Y. R. *et al.* Characterization and Pathogenic Significance of *Vibrio vulnificus* Antigens Preferentially Expressed in Septicemic Patients. *Infect. Immun.* **71**, 5461–5471, DOI: 10.1128/IAI.71.10.5461-5471.2003 (2003).

7. Kim, H. U. *et al.* Integrative genome-scale metabolic analysis of *Vibrio vulnificus* for drug targeting and discovery. *Mol. Syst. Biol.* **7**, 460, DOI: 10.1038/msb.2010.115 (2011).

8. Edgar, R. C. MUSCLE: Multiple sequence alignment with high accuracy and high throughput. *Nucleic Acids Res.* **32**, 1792–1797, DOI: https://doi.org/10.1093/nar/gkh340 (2004).

9. Edgar, R. C. MUSCLE: A multiple sequence alignment method with reduced time and space complexity. *BMC Bioinforma.* **5**, DOI: https://doi.org/10.1186/1471-2105-5-113 (2004).
